# 

**Published:** 1898-09

**Authors:** 


### CONTENTS.

COMMUNICATIONS.

Bacteriology ....................................................................401
Only a Baby Tooth—Abstract ......................................................412

PROCEEDINGS.

Proceedings of Tri-State Dental Meeting, Held at Put-in-Bay, June 21, 22 and 23, 1898..............................................................417

SELECTIONS.

Worry as a Cause of Indigestion..................................................438
Farinacea in the Diet of Later Infancy..........................................439
Infantile Mortality................................................................440
Marriage Laws.....................................................................440
Aged Parents......................................................................441
Rumination in Man.................................................................441
Music as a Sedative in Neuralgia..................................................442
Silver Citrate.....................................................................442
Tuberculosis of the Salivary Glands...............................................443
Cement for Mending Rubber Goods..................................................443
To Mend Broken Mortars............................................................443
Effect of Alcoholism Upon Paternity...............................................443
Cleanliness and Health............................................................444
Formaldehyde......................................................................444
Shock.............................................................................444
Americans Use Medicines...........................................................445
The Thumb and the Brain...........................................................445
Bone Grafting.....................................................................445

NOTICES.

The National Association of Dental Examiners.......................................445

EDITORIAL.

Why Did He Say This?..............................................................446

OBITUARY.

Dr. H. P. Southwick...............................................................448

